# Providers' Perspectives on Case Management of a Healthy Start Program: A Qualitative Study

**DOI:** 10.1371/journal.pone.0154668

**Published:** 2016-05-05

**Authors:** Imelda K. Moise, Peter F. Mulhall

**Affiliations:** 1 Department of Geography, University of Miami, Coral Gables, Florida, United States of America; 2 School of Social Work, Center for Prevention Research and Development, University of Illinois at Urbana-Champaign, Champaign, Illinois, United States of America; TNO, NETHERLANDS

## Abstract

Although Healthy Start case managers recognized the benefits of case management for facilitating optimal service delivery to women and their families, structural factors impact effective implementation. This study investigated case managers' views of 1) the structural challenges faced in implementing case management for program participants, and 2) possible strategies to enhance case management in medical home settings. Two focus groups were conducted separately with case managers from the four program service sites to gain insight into these issues noted above. Each group was co-facilitated by two evaluators using a previously developed semi-structured interview guide. The group discussions were audio recorded and the case managers' comments were transcribed verbatim. Transcripts were analyzed using thematic analysis, a deductive approach. Data were collected in 2013 and analyzed in 2015. Case managers are challenged by externalities (demographic shifts in target populations, poverty); contractual requirements (predefined catchment neighborhoods, caseload); limited support (client incentives, tailored training, and a high staff turnover rate); and logistic difficulties (organizational issues). Their approach to case management tends to be focused on linking Although Healthy Start case managers recognized the benefits of case management for facilitating optimal service delivery to women and their families, structural factors impact effective implementation. This study investigated case managers' views of 1) the structural challenges faced in implementing case management for program participants, and 2) possible strategies to enhance case management in medical home settings. Two focus groups were conducted separately with case managers from the four program service sites to gain insight into these issues noted above. Each group was co-facilitated by two evaluators using a previously developed semi-structured interview guide. The group discussions were audio recorded and the case managers' comments were transcribed verbatim. Transcripts were analyzed using thematic analysis, a deductive approach. Data were collected in 2013 and analyzed in 2015. Case managers are challenged by externalities (demographic shifts in target populations, poverty); contractual requirements (predefined catchment neighborhoods, caseload); limited support (client incentives, tailored training, and a high staff turnover rate); and logistic difficulties (organizational issues). Their approach to case management tends to be focused on linking clients to adequate services rather than reporting performance. Case managers favored measurable deliverables rather than operational work products. A proposed solution to current challenges emphasizes and encourages the iterative learning process and shared decision making between program targets, funders and providers. Case managers are aware of the challenging environment in which they operate for their clients and for themselves. However, future interventions will require clearly identified performance measures and increased systems support.

## Introduction

The high rates of preterm births, infant and maternal mortality in the United States, as well as continuing disparities in pregnancy outcomes [1–3], have prompted a number of federal and state agencies to develop programs and interventions that focus on improving the quality and continuity of care provided to women of childbearing age. Many of these efforts have focused on case management and home visiting programs to improve the health of women and families, from the postnatal period through subsequent pregnancies, to pregnancy screening for risks for complications of pregnancy, the extended use of preconception health to decrease those risks, and finally the expansion of private and public health care coverage to low-income women [4].

The achievement of an integrated system of care for high risk expectant mothers, infants, and their families is the major goal of the Healthy Start (HS) Program; a federally funded maternal and child health program that dates back to 1991 [5]. Through an integrated system of care or case management, HS programs across the United States offer coordinated and patient-centered care across the continuum of health care systems and time, providing women, children and their families the best care and support for improved maternal and infant health outcomes within communities with high infant death rates. Case management of HS participants includes 1) assessments to identify health needs and barriers to care, 2) development of an individualized care plan, 3) referrals to needed services, 4) advocacy to overcome barriers, and 5) follow-up to ensure that needed services are received. Key to effectiveness case management is the role of the case manager [6].

Health providers and their response to service delivery have long been associated with both negative and positive referral to and uptake of interventions [7, 8]. In the HS program, assessments of consumer satisfaction by and perceptions of program clients have been carried out but we know little about case managers’ views of and barriers to implementation of program services, and how these affect service provision at their medical homes as well as ability to implement coordinated care and uptake of services. Several consumer-satisfaction studies of HS services have found increased access to coordinated care and positive health outcomes for families (e.g., health care utilization, family functioning, satisfaction, and finances) [9–13]. However, one study of program staff's perceptions of program service components (e.g., outreach, case management, health education) and influence on intermediate outcomes found poor service availability and limited funding as barriers to service provision [14]. Interventions studies in other settings also confirmed high staff turnover among HS implementation barriers [15, 16].

At the same time, program implementation is influenced by levels of organization support, financial resources and adequate local capacity to use resources effectively [17] and to build needed stakeholder partnerships [18]. It’s also well-documented that case management presents a unique set of challenges to case managers when responding to the complex health care and social needs of program clients.[14, 19–23] How common these challenges are across HS grantees or how they evolve over time or are resolved remain relatively unknown. The Chicago HS Program is no exception. In addition to conducting annual local HS-impact evaluations to assess birth outcomes, client health behavior and their knowledge and perceptions since 2005, in 2011 program administrators also wanted to evaluate the state and role of organizational capacity (e.g., resources and skills) across its four federally qualified service sites on the provision of case management and program implementation. This also provided a unique opportunity to develop quality improvement guidelines to implement interventions across the four perinatal periods (preconception and interception, postpartum, prenatal, and parenting). The purpose of the current study is to examine case managers, health educators and their supervisors’ views on the structural challenges faced in implementing case management of a HS program in an urban area that is undergoing rapid population shifts including changing demographics and gentrification, and identifies possible strategies to enhance case management in medical home settings in Chicago.

## Materials and Methods

### Study setting

The study was conducted in the City of Chicago with the Chicago HS Program. The goal of the Chicago HS Program is to improve access to quality and maternal and child health services in order to reduce the high rate of infant mortality and minimize the racial disparity that exists in the project area. The project utilizes four medical centers to provide a comprehensive array of social and health services to program participants, mainly women of child bearing age and their young children ages 0–2 years residing in six Chicago community areas that are among the most “at-risk” in the City for low birth outcomes. Since inception in 1991, services have been provided in a culturally and socially appropriate manner. By creating a “one stop” facility for primary health care needs, all of the federally required Core Services and Core Systems for the project are integrated and synergistically enhanced, and paired with the provision of other services not funded by the project, including primary health care for family members, postpartum home follow-up, asthma, diabetes and other locally dictated services.

The four project family centers represent a collaborative effort between a community-based case management/social service agency and a primary health care center. This unique collaboration between an organization with expertise in the provision of social and case management services and a medical home that provides high quality preventative services overcomes many barriers to traditional service delivery. It ensures access to comprehensive, coordinated community-based care. However, because the Health Resources and Services Administration (HRSA) requires delivery of program services to residents of approved project areas or neighborhoods [24], the “targeted catchment areas” for the Chicago HS program pose challenges to case managers’ ability to implement the program. One hindrance is the “continuously changing social and political climate in which the program operates, such as demographic shifts in target populations, gentrification, the lack of available services, growing rates of poverty, State Medicaid eligibility criteria and immigration laws”[14]. Unlike the average resident, project participants tended to live below the poverty level, receive public aid, and not graduate from high school.

### Study Design

Qualitative analysis through focus groups was used to gain insights into case manager’s perceptions on challenges faced and strategies to enhance case management. A qualitative research strategy explicitly using group interaction was chosen to produce data and allow deeper insights into the problem [21, 25, 26].

### Research Participants

All the 22 program case-manager supervisors, health educators and managers from the four project family centers were recruited to participant in the study in fall 2012 and spring 2013 from an email list obtained from administrators. A total of 19 participated in the focus groups (four to seven participants at each site including 10 case managers, 3 health educators and one health therapist, and 6 supervisors. Case managers were interviewed separately from supervisors to allow case managers to freely discuss their challenges and emotions without fear [21, 25, 26]. The family centers have similar organizational structure, and target populations.

### Procedure

All materials and study protocols were reviewed and approved by the Institutional Review Board of the University of Illinois at Urbana-Champaign. This institutional review board also approved the consent procedure used in the study; with a waiver of the requirement for signed consent due to the anonymous nature of the survey (participants read a consent screen before participating). The focus groups were led by two experienced moderators, evaluation moderators with over ten years of experience in qualitative research (including facilitating focus groups), to ensure effective participation by all focus-group members. Moderators posed a series of open-ended questions using a semi-structured interview guide regarding views of the structural challenges faced in implementing case management of an urban HS Program, with a specific focus on organizational capacity (e.g., resources and skills) in the provision of case management and implementation of program interventions. The focus group guide is attached as S1 Focus Group Guide.

Focus groups lasted between 60 to 90 minutes, and participants were provided with lunch for their participation. Based upon participant consent, audio was recorded by the moderators and other notes taken. Further, participants filled out a brief survey of their demographic information, years of service, primary area of training and the nature of their service site. The focus-group discussions were audio recorded and transcribed verbatim. Transcripts were reviewed by facilitators prior to analysis to help fill in portions unclear to the transcriptionist.

### Data Analysis

Transcripts were analyzed using a deductive approach based on Braun and Clarke’s proposed five phases of thematic analysis [27], to highlight themes and issues discussed by case managers and their supervisors in both focus groups while documenting divergent themes [28]. First, interview transcriptions were read for familiarity. Second, initial codes were generated. Third, we searched for emergent themes. Fourth, we reviewed themes, and lastly we defined and named themes while ensuring that the codes accurately captured the respondent’s meaning. Inter-rater reliability and qualitative rigor was ensured by subjecting 25% of the raw data to review and coding by two independent reviewers, and the researchers further checked by presenting the study themes to a consortium and incorporating feedback into the analysis.

## Results

Data on the 19 participant are summarized in Table 1. All but one staff participant were female, and the majority were Hispanic or Latino and new to the program. In terms of education, all but three had a bachelor's degree or higher (9 Master’s and 7 Bachelor’s degrees).

**Table 1:** Focus group participant profile (n = 19).

Provider Characteristics	Frequency
**Gender**	
Female	18
Male	1
**Ethnicity/Race**	
White, Non-Hispanic	1
Black or African American	4
Hispanic or Latino	9
Asian	3
Unspecified	2
**Job Title**	
Supervisor (e.g., Manager, Senior Manager, Director)	6
Case manager	11
Health Educator	1
Unspecified	1
**Highest degree level of school**	
Some college, no degree	1
Associate degree (for example: AA, AS)	1
Bachelor’s degree (for example: BA, AB, or BS)	7
Master’s degree (for example: MA, MS, MSW) or higher	9
Unspecified	1
**Primary area of study and emphasis**	
Business	1
Health Care/Public Health Nursing	2
Maternal Health	1
Social work / Psychology	8
Substance Abuse/Domestic Violence	1
Unspecified	1
**Years of service**	
Less than a year	1
1–2 years	9
3–5 years	2
6–9 years	3
Unspecified	4
**Languages spoken**	
English	16
Spanish	9
Chinese language (e.g., Cantonese, Mandarin)	3

### Focus Group Themes

Table 2 shows the two themes that emerged in all of the focus groups:1) issues specific to structural determinants (e.g., organization and work environment), and (2) organizational determinants (e.g., organizational supports and skill levels) that have an impact on the case manager’s ability to implement efficient case management.

**Table 2:** Focus group positive themes and things to improve on.

**Theme**	**Positive Themes**
	**Organization and Work Environment**
**Structural Determinants**	Staff is motivated, knowledgeable, has skills and abilities to connect participants to various services.
	Staff is hired specifically to conduct program activities.
	Staff feels involved in the day-to-day decision-making process in regards to program participants.
	Everyone is encouraged to show leadership for project activities within their jobs—aggressive follow up to ensure client benefit from referral and postpartum services.
	Case managers value supervision and see it as supportive and constructive.
	**Outreach and Recruitment**
	Word of mouth (peers) and referral are powerful outreach tools.
	Home visitations are helpful.
**Things to Improve On**	
**Theme**	**Organization and Work Environment**
**Structural Determinants**	Lack of bus transfer cards to and from the family centers, and to referrals for specialized care (e.g., mental health services for children).
	Lack of incentives related to other prenatal services to participants e.g., bassinets, presents recruitment and participant retention challenges.
	Lack of specialized care for program participants (including behavioral/mental health, substance abuse, dental, ultrasound).
	**Outreach and Recruitment**
	Continuously changing social and political environment.
	Predefined catchment areas and high caseload.
	**Gaps in Provided Prenatal and Other Services**
	High staff turnover rate, requiring supervisors to maintain a caseload and impeding supervisory and reporting role.
	Staff needs training on how to conduct effective trainings.
	**Supports and service site’s skill levels for evaluation**
**Organizational Determinants and Other Enabling Factors**	Lack of standardized performance measures.
	**Need for Case manager training**
	Lack of consistency in the provided training.
	Need for more specially- tailored training, e.g., on child disorders.
	**Partnerships and consortium meetings for clients**
	Lack of bus transfer cards to consortium meetings, client engagement, child care, language barriers and fixed meeting locations.
	**Reporting requirements and State’s CMIS database**
	A high number of required reports, too many data entry screens and reporting assessment forms that repeat the same information.

### Challenges Specific to Structural Factors

#### Organization and work environment

Case managers identified work-group support, challenging work, creativity, organizational encouragement and internal communication as important positive work-environment factors. Work conditions and involvement in implementing core services was perceived as the most conducive to meeting the complex needs of program participants and allowing case managers to be productive. Thus, one case manager at a family center characterized case management as anticipatory and therefore affording him the autonomy to intervene directly. He perceived this a contributor to positive outcomes, including better decision making regarding client care services and implementation of core services. Case managers are forced to set priorities and make timely decisions on linking clients to adequate and timely services, built around implementation of core services, as one reviewer summed up:

*The typical Healthy Start person I encounter*, *maybe a young mother*, *first time mother*, *probably no family—no family support*, *no intentions of being pregnant*, *and probably don’t have a clue on what she’s going to do with the pregnancy*. *So*, *by being the case manager and meeting this mother*, *we kind of develop a plan on what to do with this pregnancy*. ….*and I think that is the difference with Healthy Start*.

Another participant affirmed the importance of autonomy:

*I like to think that if I wasn’t here and had the independence to intervene immediately*, *then that baby probably wouldn’t stand a chance*.

Another case manager emphasized the importance of having a supportive work group that allows case managers to consult and collaborate on an ongoing basis.

*I think my co-workers are very resourceful*. *I can go to them and they’ll help me in getting things done and preventing mistakes and getting clients linked to services quickly*.

One supervisor, when asked to explain how they deal with some case managers’ employment-related issues, said:

*As the supervisor of case managers*, *whatever problems that we have*, *we tackle them as a team*. *It is more or less*, *it’s not a weekly meeting*, *but it’s a case conference*. *So we case conference a lot*.

Case managers also appreciated supervisors’ openness and receptivity to new ideas, flexibility and accessibility.

*She has an open-door policy*, *so if we feel we have to have a meeting with her alone she is good about giving us that time*. *We also meet once a month to discuss complicated cases*.

However, some of the case managers and supervisors reported concerns on the new requirement for graduate level education for case managers and the state/program’s ability to offer competitive compensation packages, and attract those willing to work in underserved and stressful neighborhoods. For instance, there was uncertainty on the degree qualification—an advanced degree in maternal and child health or on-the-job training for organization-specific responsibilities, as expressed by this case manager:

*I know that home visiting is being looked at overall*, *but what does qualify mean*? *Does it mean a person that has a Ph*.*D*. *in maternal child health*?

Several were skeptical. For example, one participant said: *“not everyone can be successful at performing this job;”* another commented *“it takes a special person to do this type of work*.

This view was echoed by another participant who said that “*Ph*.*D holders are not going to want to work with a high risk person at the salary rate*, *in the homes*, *in neighborhoods where people are*.*”* The comment below sums up the general feeling that the job of case manager has limited financial rewards and takes a special person:

*To leave the comfort of your office and meet someone on their own—in their neighborhoods—and if you’ve seen these neighborhoods*, *we’re in the worst neighborhoods*. *There are guns*, *drugs*, *gangs; we don’t get any police escorts*, *any cell phones*, *anything*. *So*, *you have to have a need to help people or not be afraid*.

#### Outreach and Recruitment

Strategies used for outreach and recruitment varied greatly across all family centers. However, most case managers indicated that word of mouth (peers) and referral were the most powerful outreach tools. Thus, being located in a federally-qualified health center (FQDC) gave case managers’ access to patient records from the center and Medicare patients from *Cornerstone*, the State’s Case Management Information System (CMIS), and therefore improved new client recruitment, referral and retention of existing ones. Below are some responses to the question: that asked; “How do you recruit clients into the HS program?”

*Because we a federally-qualified health center*, *our clientele comes within our organization…*…. *We also have access to Medicare clients that would be assigned in Cornerstone; we have clients that don't come here*.

*I've had quite a few clients bring a client to me*. *Their friend or their family member had told them about the program*.

#### Case managers’ work load, poverty, homelessness and gentrification

On views on client recruitment and retention, many case managers were unsure about the contractual conditions and impacts of case load and challenges of working with predefined catchment areas. This included potential impacts on day-to-day tasks—including home visits—of a mandated caseload of 40 pregnant women per case manager per month.

A review of contractual conditions revealed that the Health Resources and Services Administration (HRSA) requires delivery of program services to residents of approved project areas [29] and reimburses programs based on caseload. In all four family centers, concerns were raised over the continuously changing social and political environment in which they operate, especially the rapid demographic shifts in target populations in Chicago neighborhoods due to gentrification [30, 31]. According to case managers, gentrification has displaced low income residents (target program participants), and along with the fixed service areas exacerbated challenges on outreach efforts, as expressed by these case managers:

*Our boundaries stay the same but the population moved out*.

*Lots of clients moved out of the area because the neighborhoods are becoming expensive to live in*, *so some of those clients*, *even though they have high risk*, *we cannot really enroll them because they’re out of the target area*.

Although there have been efforts to expand outreach efforts to meet such demographic shifts in target populations in Chicago, not all were successful at meeting the caseload requirements of 40 pregnant women per month per case manager. For example, one case manager noted,

*We are supposed to have 40 pregnant women each month*, *so right now*, *because of our struggle with finding clients in the area*, *I have 16 pregnant women and the rest are infants or children*.

In addition, the growing rates of poverty and homelessness among target neighborhoods, and the highly mobile and transient nature of clients has undermined client recruiting and home visits. Some participants expressed the belief that the city of Chicago did not account for the effects of gentrification on renters who lived with friends, most of whom qualify for program services. As one supervisor put it:

*I would think that—when the government started tearing the projects down*, *I don’t think they accounted for all the people who squat in other people’s homes*. *So you give a resident a new apartment*, *but this resident had 30 families living there*, *literally*, *30 families living in her house*. *And with the new rules*, *no one but her can be on the lease*. *So*, *now*, *she can’t bring anybody with her*. *So*, *that leaves all of these families out here*.

Case managers at one service site addressed the above-described tension by operating two sites to meet caseload requirements. Others indicated retaining program clients on caseload if they moved out of the catchment area but came back to receive services.

*We have many program clients that move out of the area but if they have lived in this area*, *if they moved out*, *they let us follow them*.

While a few case managers felt that the program should allow catchment neighborhoods to expand to reflect the changing demographic shifts, most were reluctant, fearing new challenges for performing day-to-day tasks, home visitations and meeting contractual requirements. When asked, “What are some challenges that will emerge if boundaries were expanded?” one case manager responded:

*Let us say I have 40 pregnant women on my case load*. *The grant requires two face-to-face contacts with every pregnant woman a month so that is 80 face-to-face*. *Out of the 80 face-to-face*, *40 of them have to be home visits*. *That is 40 home visits and 40 office visits*, *assuming all of the clients come in here to see us … but it is not possible if the boundaries expand*.

### Gaps in Prenatal and Other Service Provision

Case managers in both focus groups generally suggested additional training for capacity building and enhanced skills, and support with training materials, to allow them to better respond to challenges emerging from the changing program demographics, notably in areas of immigration and health insurance.

*Because we work with a high immigration populations they get nervous about us going to their home and they often have trouble understanding the complex government system when applying for services*, *and why we have to conduct home visitations*. *They think we are going to report them; as such we have to clear that up right away*.

Some case managers cited problems with finding housing for program clients, and clients having problems with access to specialized care, and lacking courage to seek services, support and episodic care after hours. Clients needing specialized care are often referred.

*Sometimes it’s just even transportation*, *just getting here*. *……or being able to travel to access specialized care on behavioral health*, *dental*, *and ultrasound*.

Case managers and supervisors also bemoaned the lack of client incentives to assist in outreach and client retention, and favored the reinstating of such incentives across service sites.

### Organizational Determinants and Other Enabling Factors

#### Support and service-site skill levels for evaluation

Most case managers had mixed feelings on whether or not their service site is skilled at using data to assess program impacts (e.g. ages and stages), monitor program core services or undertake long term participant follow ups. Further, case managers spoke to the need for clearly identified performance measures, rather than just operational outputs, for monitoring program impact. Both focus groups revealed a high level of frustration caused by this lack of standardized performance measures, which makes it difficult, for example, to measure the success and impact of educational materials.

*How do you measure something which will be different*, *of course*, *in every organization*?

*Measurements*, *it's black and white*. *We’re not talking the same language*, *the same outcome*, *and the same measurements*.

When asked to indicate their level of agreement with each of the organizational support statements, a majority agreed that case managers are appropriately trained, hired specifically to implement program core services, have timely access to information about core services, and have participated in professional development. Some also agreed that staffing levels are adequate, and that there is adequate administrative support and professional development opportunities at their service sites and within the program.

#### Need for case manager training

Case managers acknowledged receiving different types of training during the course of their employment and expressed satisfaction with the on-the-job training. They, however, revealed a lack of consistency in the training provided, with some indicating knowledge gaps on important maternal and child health topics. Case managers would like to have more training tailored to child disorders (e.g., autism, Down syndrome), the Affordable Care Act, and to strategies on how to approach an outside agency. Several case managers expressed the need for training and seminars on immigration-related issues, as well.

*Many of our clients are undocumented; they don’t have access to many services that people with low income who are U*.*S*. *citizens have access to*.

#### Benefits of mandated partnerships and consortium meetings for clients

Focus-group discussions of views on and suggestions for mandated consortium meetings in terms of client benefits, case managers generally perceived building partnerships and collaborations with the community and a number of agencies as essential for delivery of care closer to communities and in linking clients to needed services. Likewise, participation in consortium meetings was believed to enhance partnerships and networks among HS family centers and maternal and child health programs, and was essential for a return to seamless, unduplicated services from multiple service providers and better outcomes around quality healthcare for women and children within local health systems.

Consortium meetings are held bimonthly (on fourth Wednesday of every other month), and each center is contractually required to send at least two client representatives to ensure that the whole community is committed to reduce infant mortality and low birth weight in target neighborhoods. They offer an opportunity for family centers to keep client families at the forefront of service delivery. Topics discussed varies, including discussing, planning, and providing unique community activities or training that strengthen and empowered perinatal health in the target neighborhoods, and contribute to efforts to establish perinatal health standards often shared throughout the neighborhoods.

However, all four family health centers also experienced challenges with engaging and sustaining client participation at consortium meetings, including poor attendance. Causes identified for the low client participating included conducting consortium meetings too early in the day for some clients, and lack of client transfer bus cards, client engagement, and child care, along with language barriers and fixed meeting location. The following quotes from case managers illustrate the client-participation challenge.

…..*for a Mom that has children*, *whether it's one or three*, *and she has to get them to school and it starts at 9*:*30; that's why we don't have a high turnout as we want to*.

*We are encouraged to bring clients but our clients don’t understand what they are talking about*. *We don’t have a translator there*.

Some case managers suggested that consortium meetings be rotated among each family center instead of having them at the same location. Others proposed the reinstating of client incentives across service sites to enhance outreach and client retention.

#### Reporting requirements and State’s Case Management Information System (CMIS)-Cornerstone

Most case managers reported problems in using the state CMIS. These include a high number of required reports in the CMIS database and being user-unfriendly—too many data entry screens, and reporting assessment forms that repeat the same information. One case manager had this to say:

*I think Cornerstone is a helpful tool*, *to keep records on what we are working on with a client*. *I just think a lot of the information is too repetitive*.

Some reported on the issue of too many screens on follow up:

*You have to enter so many screens*, *it is so easy to miss one screen and it is almost like you didn’t do the work if you didn’t enter that one screen*—(another)

A common view amongst interviewees in regards to the CMIS and data use is that this *“illustrates the apparent discrepancies in information kept at family centers and information that the state downloads from CMIS*,” and concerns that the State’s data do not reflect actual program performance.

## Discussion

This study provided insights into the challenges that supervisors and case managers face in the provision of case management to women, children and families enrolled in the Chicago HS program. Our findings revealed that numerous structural factors influence supervisors’ and case managers’ ability to more efficiently provide case-management services.

As in previous studies [32, 33], these supervisors and case managers recognized the benefits of using case management as a vehicle for overcoming system fragmentation and for facilitating optimal service delivery to women and their families, and sought to link clients to available services in a timely manner. They clearly also strove to meet the program rules, guidelines and timelines, and respond to the evolving operating environment through rapid demographic shifts and gentrification [30, 31]. Likewise the impact of case managers work load of poverty, homelessness and gentrification are mixed. On one hand, poverty, homelessness and gentrification undermines the ability of case managers to recruit and retain patients and therefore meeting the mandated caseload. On the other hand, this potentially increases the workload in terms effort per client in cases where case managers are forced to follow clients outside mandated service areas in order to meet mandated caseloads. This has forced providers to consistently balance meeting contractual service-area conditions [24] and addressing the real needs of shrinking or mobile clients. The required caseload in the predefined program catchment neighborhoods and home visits are less likely to be achieved under the current operating environment.

Our findings also help explain why inconsistent implementation under the HS program [14, 34] undermines the performance of case management, an otherwise robust and powerful model, particularly, for individuals who have multiple health needs. For instance, there remain tensions in meeting contractual client-participation requirements and addressing real challenges that undermine same participation, even as efforts to address it are undertaken. These results corroborate the ideas of Plough (1994), who suggested that “there are conflicts inherent in a model that is defined and controlled by the federal government but simultaneously calls for substantial community participation and control” [19] Further, the overall program complexity of striving to safeguard community and civic participation while trying to adhere to other contractual conditions had most case managers feeling overwhelmed. These results are also consistent with other studies which found that efficient program implementation is generally affected by organization support and adequate local capacity to use resources effectively [17, 18].

This finding is particularly important in light of studies showing that the underlying theoretical model driving how a program is put in place is associated with missed opportunities for reaching out to those in need of services [35]. In short, while restricting the provision of services to the predefined neighborhoods may meet contractual conditions, the program may not consistently reach those who need the HS program services the most. These results further support the idea of Hanks (2008), who calls for a new model that “builds on the iterative learning cycles and shared decision making of community participatory research to better address the transactional relationship between program targets, providers and funder needs [35],” than rigid adherence to the current HS models.

Training in the implementation of achievable quality outcomes and performance measures should target all case managers responsible for providing direct services to program clients. Furthermore, some studies indicate that factors other than case manager training (e.g., “the tension between the need for consistent measurement across communities and the need for flexible measures that reflect community-specific adaptations and conditions” [36]) may be more important predictors of delivery of interventions. These findings provide further support for the need for performance measurements in general which clearly delineate target benchmarks and definitions for performance improvement.

Another major concern was the CMIS, in particular the perception by case managers that it is not adequately user friendly or customized to local needs. Some program staff reported using a workaround plug-in, a report writing module/add-on to the CMIS, but because no training had been provided, most program staff was unable to use it. These results were also observed in our previous study [21].

This study was exploratory and bears the limitations inherent in such research, such as the inability to generalize given the small sample size. While the sample size of 19 respondents may appear small, it covers nearly all Chicago program employees who work directly with program clients in the delivery of all program components in the four service centers participated in the focus groups, and the number is reasonable for case-study qualitative research. The skewed gender representation (only one man out of 19) is inherent in the provider population. Further, most participants were new to the program (1 to 2 years) and virtually all Hispanic or Latino. This potentially limits the diversity of views and excludes those of other ethnic groups. In addition, our study used predefined themes adapted from Hanusaik and colleagues’ [37] conceptual model. In truth, this theory contains a number of additional determinants that our study was not designed to evaluate. However, despite these limitations and the exploratory nature of this study, it offers insights for a nuanced understanding of the challenges that many HS case managers encounter or are likely to encounter as they implement case management to link women and children to needed services, and opens avenues for targeted interventions to resolve some of the issues.

In summary, this small qualitative study demonstrated that case managers are sensitive to contractual conditions, although they operate in an environment that is undergoing rapid population shifts, changing demographics and gentrification. New approaches may help alleviate frustrations relating to structural factors that undermine effective implementation of case management in the program. In addition, a system that emphasizes quality improvement and the newly transformed Healthy Start 3.0 currently underway should help.

## Supporting Information

S1 Focus Group Guide Guiding questions used during focus group discussions. (DOCX)
