# Correction: Providers' Perspectives on Case Management of a Healthy Start Program: A Qualitative Study

**DOI:** 10.1371/journal.pone.0157803

**Published:** 2016-06-13

**Authors:** The PLOS ONE Staff

The Abstract is partially duplicated in the published article. The correct Abstract is:

Although Healthy Start case managers recognized the benefits of case management for facilitating optimal service delivery to women and their families, structural factors impact effective implementation. This study investigated case managers' views of 1) the structural challenges faced in implementing case management for program participants, and 2) possible strategies to enhance case management in medical home settings. Two focus groups were conducted separately with case managers from the four program service sites to gain insight into these issues noted above. Each group was co-facilitated by two evaluators using a previously developed semi-structured interview guide. The group discussions were audio recorded and the case managers' comments were transcribed verbatim. Transcripts were analyzed using thematic analysis, a deductive approach. Data were collected in 2013 and analyzed in 2015. Results showed that case managers are challenged by externalities (demographic shifts in target populations, poverty); contractual requirements (predefined catchment neighborhoods, caseload); limited support (client incentives, tailored training, and a high staff turnover rate); and logistic difficulties (organizational issues). Their approach to case management tends to be focused on linking clients to adequate services rather than reporting performance. Case managers favored measurable deliverables rather than operational work products. A proposed solution to current challenges emphasizes and encourages the iterative learning process and shared decision making between program targets, funders and providers. Case managers are aware of the challenging environment in which they operate for their clients and for themselves. However, future interventions will require clearly identified performance measures and increased systems support.

The publisher apologizes for the error.
