# Supplementary material for: Providers' Perspectives on Case Management of a Healthy Start Program: A Qualitative Study
Source: PLoS One. 2016 May 5;11(5):e0154668. doi: 10.1371/journal.pone.0154668 (PMC4858192; doi:10.1371/journal.pone.0154668)
Supplement: S1 Focus Group Guide — (DOCX) [file pone.0154668.s001.docx]

**Healthy Start Professional Staff Focus Group Questions**

**Thank you for taking time to talk with us today.**

**(Read Confidentiality Statement/Consent to record interview)**

***Background***

1. Let’s do a quick round of introductions. Can each of you tell the group your title/role and how long you have been working with the Chicago Healthy Start Initiative and with Family Health Center?
2. Next, we’d like to hear about your organization and work environment;
   - Given your experiences, describe what the Health Center does for the community?
   - Which Chicago community areas do you serve?
   - What is your case load like? Describe your typical work day?
   - What factors help you do your job?
   - What factors are obstacles to doing your job?
   - What about local, state and agency policies and procedures?
   - What is the approximate racial breakdown of your workforce? Clients?
   - Do you carry out home visits with your clients? How many home visits do you carry out on average? Monthly? For prenatal case management? For Postnatal case management?
   - What are your working conditions like? Do you work in an office or out in the field?
   - What’s the general work environment like for employees at the Family Health Center in regard to having the necessary support to be able to get your job done in the best way possible? Probe:
     - Some key issues we are interested in include being included in decision making, having the proper autonomy and authority to make decisions, having access to the information you need, and having support from your management.
   - How would you rate the quality of the supervision, management, and leadership you receive at the Family Health Center? And the Healthy Start Chicago Initiative?
     - Why do you rate it that way?
     - Do managers or supervisors understand and appreciate the job that you do and the challenges you face? Why or why not?
     - What about agency policies and procedures?
3. What are the reporting requirements? Probe: How often do you report? Monthly? Quarterly? Annually? Perinatal depression? Ages and stages reports for infants, interconeptional management? What are you capturing in each of these reports?
4. We know you have conducted trainings with your clients. Could you kindly tell us what trainings you have implemented? What was participation like?
5. What additional training would help you do your job more effectively?
6. In thinking about co-workers who have recently left, what do you think were their reasons for leaving?
7. If the Family Health Center and the Healthy Start Chicago Initiative could do only two things to improve the organizational and work environment at the center and project level, what should they be?
8. ***The following questions are about enrollment/eligibility of participants into the Healthy Start Chicago Initiative:***
   - Can you describe the initial assessment process for participants’ eligibility and enrollment into the Healthy Start Chicago Initiative for us?
   - Share with us your impressions of the initial assessment process thus far. How has it been received by your clients?
   - Do you think the current initial assessment process encourages or discourages pregnant women to enroll in the initiative? Why?
   - Which assessment risk factors and local contributing factors (direct and indirect) that effect birth weight, infant mortality and cost are you mostly likely to use in determining a participant’s initial eligibility/enrollment into the Healthy Start Chicago Initiative? [Distribute Form 707G-Asssessment]
   - Any final thoughts or comments about the initial assessment process?
9. ***Now, we want to ask you about your implementation of the five (5) Core Services:***

The primary objective of the Healthy Start Chicago Initiative is to improve birth outcomes for at risk pregnant women and the recruitment/enrollment of “hard” to reach, high risk population” from project target areas.

1. How do you identify/recruit “hard” to reach, high risk population served at Health Family Center?
2. What are some barriers and challenges that you expnce related to the Implementation of successful outreach/enrollment? Possible prompts:
   1. Budgets limitations
   2. Significant others
   3. Transient nature of high-risk women in target area
3. What interventions do you believe have an impact on the successful outreach/enrollment? Possible prompts:
   1. Transportation
   2. Interface with family case management
   3. Interface with other agencies/programs
   4. Incentives
4. On a scale of 1 to 5, what are the most important Core Services related to achieving the following intermediate objectives:
   1. First trimester prenatal care
   2. Adequate prenatal care
   3. Well-child exams (i.e. EPSDT)
   4. Required immunizations
   5. Positive health behaviors
5. What are the type and intensity of services provided and/or referred to for Healthy Start clients?
6. In your view, what is the risk profile of the “typical” Healthy Start pregnant participant Family Health Center? Or how would you describe the “hard” to reach, high risk” population served by the Healthy Start Chicago Initiative at your Family Health Center? Possible prompts:
   1. Race? Age? Risk factors?
7. In your view, what are the most reported risk factors for pregnant participants at Family Health Center? Possible prompts:
   1. Diseases that affect pregnancy?
   2. Homelessness/Temporary Housing?
   3. Low education attainment? HIV or repeated STD?
   4. Previous preterm birth?
8. What areas if any do you believe your agency could improve on? E.g. enrollment, post-partum, referrals?
9. ***Let’s talk about your project’s level of implementation of interconceptional Care (ICC)?***
   - In your view, how many of your clients complete an ICC or are referred to one?

- What are some barriers and challenges that you expnce related to the implementation of ICC? Possible prompts: Short time frame to develop relationship with pregnant women who enroll in last trimester; DHS policy that emphasizes enrolling pregnant women over retaining interconceptional women in caseload
  - What interventions do you believe have an impact on the successful ICC?

1. We understand there are some challenges with appropriate documentation of events and data entry into Cornerstone.
   - How have you addressed that in your program?
2. What additional training and technical assistance do you think would be helpful to better prepare you for implementation of the 5 Core Services?
3. As we wrap up, I’d like to ask if there’s anything about the implementation of the 5 Core Services and your overall work with the Healthy Start Initiative that we didn’t talk about today that you’d like to mention or discuss.

**Five (5) Core Services**

1. ***Outreach and recruitment*** of high-risk pregnant women: Pregnant women identified from target areas and; Women who initiate prenatal care in 1^st^ trimester
2. ***Case management****:* Eligible high-risk pregnant/postpartum women/infants enrolled in the program and WIC; Adequacy of prenatal care; Referrals made and kept for pregnant, child interconceptional participants; Linkage to a medical home (women and children-0-2 years old); Pregnant participants who deliver at level III or level II+ hospitals; Participants who complete postpartum visit; Children (0-2) who are up-to-date with immunizations and; Children (0-2) who are up-to-date with EPSDT exams.
3. ***Health Education of pregnant and interconceptional participants:*** Pregnant women who self- report reduction/cessation in smoking; Pregnant women who self- report reduction/cessation in substance abuse during pregnancy; Interconceptional participants that self-report breast feeding of infants; Interconceptional participants that self-report using family planning services; Interconceptional participants with more than 18 months between pregnancies)
4. ***High Risk Interconceptional Care* of pregnant participants and interconceptional women**: women who complete a post-partum visit; Women who receive in interconceptional services; Women linked to a medical home; Women whose pregnancies occur within 24 months of a previous birth; Women who receive family planning services in the post-partum period)
5. ***Prenatal Depression:*** Women who deliver and are screened for depression; Women who are screened for depression (EPDS) and are referred for diagnosis/treatment; Women who referred for and received treatment/consultation for depression.
